# Intravital Metabolic Autofluorescence Imaging Captures Macrophage Heterogeneity Across Normal and Cancerous Tissue

**DOI:** 10.3389/fbioe.2021.644648

**Published:** 2021-04-20

**Authors:** Tiffany M. Heaster, Alexa R. Heaton, Paul M. Sondel, Melissa C. Skala

**Affiliations:** ^1^Department of Biomedical Engineering, University of Wisconsin–Madison, Madison, WI, United States; ^2^Morgridge Institute for Research, Madison, WI, United States; ^3^Department of Human Oncology, University of Wisconsin–Madison, Madison, WI, United States; ^4^Department of Pediatrics, University of Wisconsin–Madison, Madison, WI, United States

**Keywords:** fluorescence lifetime imaging, autofluorescence, cellular metabolism, intravital microscopy, macrophages

## Abstract

Macrophages are dynamic immune cells that govern both normal tissue function and disease progression. However, standard methods to measure heterogeneity in macrophage function within tissues require tissue excision and fixation, which limits our understanding of diverse macrophage function *in vivo*. Two-photon microscopy of the endogenous metabolic co-enzymes NAD(P)H and flavin adenine dinucleotide (FAD) (metabolic autofluorescence imaging) enables dynamic imaging of mouse models *in vivo*. Here, we demonstrate metabolic autofluorescence imaging to assess cell-level macrophage heterogeneity in response to normal and cancerous tissue microenvironments *in vivo*. NAD(P)H and FAD fluorescence intensities and lifetimes were measured for both tissue-resident macrophages in mouse ear dermis and tumor-associated macrophages in pancreatic flank tumors. Metabolic and spatial organization of macrophages were determined by performing metabolic autofluorescence imaging and single macrophage segmentation in mice engineered for macrophage-specific fluorescent protein expression. Tumor-associated macrophages exhibited decreased optical redox ratio [NAD(P)H divided by FAD intensity] compared to dermal macrophages, indicating that tumor-associated macrophages are more oxidized than dermal macrophages. The mean fluorescence lifetimes of NAD(P)H and FAD were longer in dermal macrophages than in tumor-associated macrophages, which reflects changes in NAD(P)H and FAD protein-binding activities. Dermal macrophages had greater heterogeneity in optical redox ratio, NAD(P)H mean lifetime, and FAD mean lifetime compared to tumor-associated macrophages. Similarly, standard markers of macrophage phenotype (CD206 and CD86) assessed by immunofluorescence revealed greater heterogeneity in dermal macrophages compared to tumor-associated macrophages. Ultimately, metabolic autofluorescence imaging provides a novel tool to assess tissue-specific macrophage behavior and cell-level heterogeneity *in vivo* in animal models.

## Introduction

Macrophages serve several roles in tissue maintenance and inflammatory response in both normal and diseased tissues (Gordon and Plüddemann, 2017). There is limited understanding of functional differences in macrophages across these tissue sites and of macrophage involvement in tissue dysfunction. Additionally, macrophage plasticity results in multiple unique functional phenotypes, presenting further challenges for assessing macrophage function (Shapouri-Moghaddam et al., 2018). Standard functional assays (e.g., flow cytometry, PCR, ELISA, and histology) require destructive sample preparation, which limit assessment of macrophage behavior *in vivo* (Chattopadhyay et al., 2014). Non-destructive methods of monitoring macrophage function are needed to better understand macrophage plasticity and the role of macrophages in regulating tissue homeostasis and disease pathogenesis.

Tissue niche conditions, such as cytokine/chemokine secretion, nutrient availability, and collagen organization, drive macrophages to adopt various functional phenotypes (Epelman et al., 2014; Lavin et al., 2014; Sridharan et al., 2019; Sapudom et al., 2020).Generally, tissue-resident macrophages serve to protect against invading pathogens and promote tissue repair, independent of tissue origin (Gordon and Plüddemann, 2017). Previous studies have shown that functional gene expression differs between macrophages originating from distinct tissue sites, and these macrophages can be functionally reprogrammed upon trafficking to other tissues (Lavin et al., 2014). Additionally, tumor-infiltrating macrophages are specifically directed to promote tumor progression by signaling tumor cell proliferation, motility, and angiogenesis while suppressing further immune recruitment (De Palma and Lewis, 2013). However, comparisons between distinct macrophage populations across normal and diseased tissue sites are limited due to constraints of functional assays (De Palma and Lewis, 2013; Panni et al., 2013).

Changes in macrophage phenotype and function have been directly correlated to their metabolic demands (Stout et al., 2005; De Palma and Lewis, 2013; Panni et al., 2013). Macrophages undergo metabolic switching between oxidative and glycolytic metabolism to fuel anti- or pro-inflammatory processes, respectively (Diskin and Pålsson-McDermott, 2018). Energy demands of tissue-resident macrophages are regulated by the tissue microenvironment, though characterization of macrophage metabolic profiles across specific tissues are limited (Caputa et al., 2019). Previous studies suggest that tissue-resident macrophages require stable upregulation of both glycolysis and oxidative phosphorylation to promote survival and proliferation in tissue (Van Den Bossche et al., 2015). Tumor-infiltrating macrophages are primarily characterized by an anti-inflammatory phenotype relying on fatty acid oxidation and oxidative phosphorylation (Panni et al., 2013). Conversely, highly glycolytic macrophage populations have also been observed invading tumors, demonstrating metabolic heterogeneity associated with tumor-infiltrating macrophages (Vitale et al., 2019). Tools for monitoring macrophage metabolic changes *in vivo* may better visualize this heterogeneity and inform on cell-level behavior of macrophage populations and interactions with the tissue microenvironment.

Two-photon microscopy of reduced nicotinamide adenine dinucleotide (phosphate) (NAD(P)H) and oxidized flavin adenine dinucleotide (FAD), or “metabolic autofluorescence imaging,” monitors metabolic activity on a single-cell level. NAD(P)H and FAD are an electron donor and acceptor, respectively, involved in metabolic reactions across numerous pathways (Chance et al., 1979; Walsh et al., 2013; Blacker and Duchen, 2016). NAD(P)H and FAD fluorescence intensities report on their respective abundance within cells (Chance et al., 1979; Blacker and Duchen, 2016). Furthermore, the optical redox ratio, defined as the ratio of fluorescence intensity of NAD(P)H to FAD, provides a quantitative measurement of the relative oxidation-reduction state of individual cells (Lakowicz et al., 1992; Georgakoudi and Quinn, 2012; Walsh et al., 2013; Blacker and Duchen, 2016). The fluorescence lifetimes of free and protein-bound NAD(P)H and FAD are distinct and can be recovered with a bi-exponential decay model (Lakowicz et al., 1992; Georgakoudi and Quinn, 2012; Quinn et al., 2013; Walsh et al., 2013). Therefore, fluorescence lifetime imaging microscopy (FLIM) of NAD(P)H and FAD reflects the protein-binding activities of these metabolic co-enzymes in the cell. Previous studies have demonstrated that *in vivo* tumor-associated macrophages have high FAD intensities that can discriminate these macrophages from other cell types (Szulczewski et al., 2016; Li and Liu, 2018). However, tissue-specific macrophage metabolism and the metabolic heterogeneity of macrophages within a tissue have not been thoroughly characterized *in vivo* due to a lack of appropriate tools. NAD(P)H and FAD autofluorescence have been previously used to distinguish metabolically diverse cell sub-populations (Kilarski et al., 2013; Ghesquière et al., 2014; Tong et al., 2015; Walsh and Skala, 2015; Alfonso-García et al., 2016; Shah et al., 2017; Heaster et al., 2018, 2020; You et al., 2018; Jones et al., 2020; Li et al., 2020; Smokelin et al., 2020), which supports the use of metabolic autofluorescence imaging to investigate macrophage heterogeneity *in vivo*. This study demonstrates that metabolic autofluorescence imaging can quantify metabolic heterogeneity between macrophages within normal and cancerous mouse tissues *in vivo*.

## Methods

### Mouse Breeding, Inoculation, and Surgery

**FIGURE 1:**
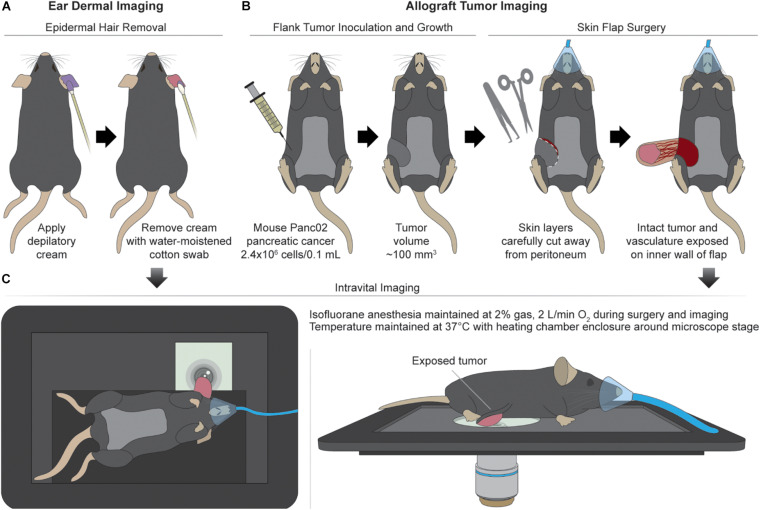
Mouse surgical preparation and intravital imaging setups. **(A)** Ear dermal imaging technique. Mouse is anesthetized and depilatory cream is applied for ∼2 min to the ventral side of the mouse ear to remove hair as a source of imaging artifact. Depilated area is cleaned with water-soaked cotton swab. **(B)** Allograft generation and imaging technique. Mouse is inoculated subdermally with 2.4 × 10^6^ Panc02 pancreatic cancer cells (per 0.1 mL) in the flank and allowed to grow to a volume of ∼100 mm^3^. After reaching target volume, mouse is anesthetized for surgical exposure of tumor. Superficial incisions are made into skin surrounding the tumor to form a flap separable from the peritoneum and body cavity. The tumor is exposed by peeling the flap away from the body while remaining intact and vascularized. **(C)** Intravital imaging procedures. For ear dermal imaging, mouse is placed on microscope stage designed with inlay to support mouse body and imaging window on which the cleared ear skin is overlaid. For tumor imaging, mouse is placed on microscope stage and skin flap with attached tumor is overlaid for imaging. Anesthesia and temperature are regulated and maintained during all imaging procedures.

Csf1r-HBEGF/mCherry and Lyz2-Cre mice were obtained from Jackson Labs and bred to generate immunocompetent progeny with mCherry-expressing macrophages (Schreiber et al., 2013). The reporter mCherry was chosen to avoid spectral overlap with autofluorescence of NAD(P)H and FAD, and to confirm the identity of *in vivo* macrophages. Intravital imaging of the mouse ear dermis was performed by adapting previously established protocols (*n* = 4 mice) (Li et al., 2012; Kilarski et al., 2013). Prior to imaging, depilatory cream was applied for ∼2 min to the dorsal side of the ear of isoflurane-anesthetized mice to remove artifacts from hair. Depilatory cream was removed quickly with a moistened cotton swab to remove cream and loose hair and the area was thoroughly cleaned to prevent chemical skin burn and avoid inflammation (Figure 1A). Additional application of PBS was performed to provide extra hydration of the cleared area. Subdermal tumors were engrafted in the same mice used for ear dermis imaging by injecting 2.4 × 10^6^ Panc02 pancreatic adenocarcinoma cells per 0.1 mL into the flanks of each mouse (*n* = 4). Panc02 cells used for subdermal injections were obtained from the NCI and were cultured in standard RPMI1640 (Gibco) + 10% FBS + 1% penicillin:streptomycin at 37°C and 5% CO_2_. Imaging commenced when tumors reached ∼100 mm^3^. Immediately prior to tumor imaging, skin flap surgery exposed flank tumors. Mice were anesthetized with isoflurane, then the skin around the tumor was cut and separated from the body cavity so that the tumor laid flat on the imaging stage while connected to the vasculature (Conway et al., 2017) (Figure 1B). Mice were placed on a specialized microscope stage for imaging (Figure 1C). For dermal imaging, a glass coverslip insert was used with PBS for tissue coupling and surgical tape to secure the tissue. For tumor imaging, an imaging window insert was used with surgical tape to secure skin flap tumors.

### Intravital Fluorescence Lifetime Imaging

Autofluorescence images were captured with a custom-built multi-photon microscope (Bruker) using an ultrafast femtosecond laser (InSight DS+, Spectra Physics). Fluorescence lifetime measurements were performed using time-correlated single photon counting electronics (Becker & Hickl). Fluorescence emission was detected using bandpass filters of 466/40 nm (NAD(P)H), 514/30 nm (FAD), and 590/45 nm (mCherry) prior to detection with GaAsP photomultiplier tubes (Hamamatsu). All three fluorophores were concurrently excited using a previously reported wavelength mixing approach (Mahou et al., 2012; Stringari et al., 2017). Briefly, the laser source tuned to 750 nm (NAD(P)H excitation) was delayed and collimated with the secondary laser line fixed at 1,041 nm (mCherry excitation) for spatial and temporal overlap at each raster-scanned focal point (2-color excitation of FAD with 750 nm + 1041 nm). All images were acquired with a 40X/1.13 NA water-immersion objective (Nikon) at 512 × 512 pixel resolution and an optical zoom of two restricting the field of view (150 μm × 150 μm) to the area of laser overlap. A daily fluorescence standard measurement was collected by imaging a YG fluorescent bead (Polysciences Inc.) and verifying the measured lifetime with reported lifetime values. NAD(P)H and FAD intensity and lifetime volumes (*z*-depths ranging from 20 to 75 μm, 5 μm *z*-steps) were acquired to sample metabolic behavior of macrophages across 3–5 fields of view and multiple depths within each tissue. The fluorescence lifetimes of free and protein-bound NAD(P)H and FAD are distinct, and these lifetimes along with their weights can be recovered with a two-exponential fit function. Therefore, fluorescence lifetime data for both NAD(P)H and FAD was fit to the following bi-exponential decay in SPCImage: *I*(*t*) = α_1_*e*^−*t*/τ_1_^ + α_2_*e*^−*t*/τ_2_^ + *C*. For NAD(P)H, τ_1_ corresponds to the free lifetime, τ_2_ corresponds to the protein-bound lifetime, and the weights (α_1_, α_2_; α_1_ + α_2_ = 1) correspond to the proportion of free and protein-bound NAD(P)H, respectively (Lakowicz et al., 1992; Georgakoudi and Quinn, 2012; Blacker and Duchen, 2016; Datta et al., 2020). Conversely for FAD, τ_1_ corresponds to the protein-bound lifetime and τ_2_ corresponds to the free lifetime (Ramanujam, 2000; Datta et al., 2020). Decays within a 3 × 3 pixel area were binned to improve signal-to-noise ratio. An instrument response function was measured with second harmonic generation (900 nm excitation) from urea crystals for input into the decay fit procedure. The following fluorescence lifetime components were calculated from the fitted model: τ_1_, τ_2_, α_1_, and α_2_ for both NAD(P)H and FAD. Mean fluorescence lifetimes (τ_m_) were calculated as τ_m_ = τ_1_α_1_ + τ_2_α_2_.

### Geodesic Reconstruction

**FIGURE 2:**
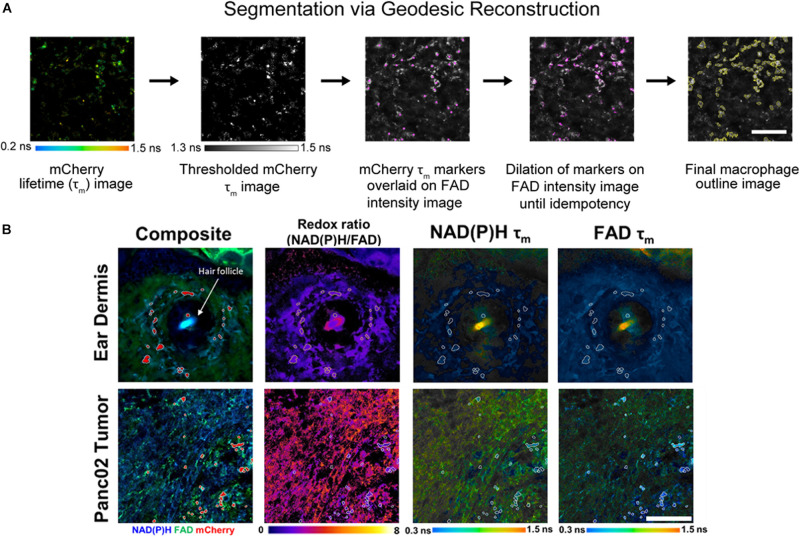
*In vivo* metabolic autofluorescence imaging and segmentation of macrophages in tumor and dermal tissue. **(A)** Geodesic reconstruction for segmenting mCherry+ macrophages. Lifetime images of mCherry were thresholded [1.3–1.5 ns, based on reported mCherry lifetime values (Gu et al., 2013; Penjweini et al., 2018; Štefl et al., 2020)] to exclude any non-specific fluorescence in the red emission channel. Thresholded images were used as a marker image to identify mCherry+ cells. FAD intensity images were used as mask images based on previous evidence of high FAD intensity in macrophages (Szulczewski et al., 2016). The marker image was overlaid on the mask image and iteratively dilated until all connected component objects in the mask image were intersected (idempotency). Cell outlines were then determined from the reconstructed masks. Scale bar = 125 μm. **(B)** Representative fluorescence images of mouse ear dermis and Panc02 pancreatic tumors. Composite intensity images visualize tissue-infiltrating macrophages (mCherry, red) with co-localized NAD(P)H (blue) and FAD (green) autofluorescence. Autofluorescence intensity and lifetime images demonstrate qualitative differences in the optical redox ratio [intensity of NAD(P)H divided by intensity of FAD], NAD(P)H mean lifetime (τ_m_), and FAD τ_m_. Ear dermis images depict a hair follicle (white arrow), which consistently exhibited increased accumulation of macrophages. Conversely, random distribution of macrophages was observed across pancreatic tumor images. Scale bar = 125 μm.

Individual cell masks were segmented using Geodesic Reconstruction (MorphoLibJ, Fiji) (Legland et al., 2016) depicted in Figure 2A and described as follows: (1) mCherry lifetime images were thresholded based on reported lifetimes (1.3–1.5 ns) to exclude any non-specific fluorescence in the red fluorescence emission channel (Gu et al., 2013; Penjweini et al., 2018; Štefl et al., 2020), (2) thresholded images were designated as marker images for identifying mCherry+ cells, (3) FAD intensity images were designated as mask images based on high FAD signal associated with macrophages (Szulczewski et al., 2016), (4) the marker image was overlaid on the mask image and was iteratively dilated until all connected component objects in the mask image were intersected (i.e., idempotency), and (5) cell outlines were then determined from the reconstructed masks. Intensity and lifetime components for each pixel within resulting masks were averaged in CellProfiler to obtain single-cell measurements. Agreement between geodesic reconstruction and manual segmentation were confirmed by Dice coefficients (Zou et al., 2004) of representative images (Supplementary Figure 1).

### Immunofluorescence Staining and Imaging

Following the final *in vivo* imaging session for each mouse (post-dermis and tumor imaging), animals were euthanized and both tumors and ear dermis were excised for immunofluorescence staining of macrophage abundance and phenotype. Excised tissues were formalin fixed and paraffin-embedded for antibody staining with a panel of fluorescent markers (CD86, CD206, and mCherry). Sections were taken 5 μm apart through the first 100 μm from the tissue surface to sample from similar tissue areas as measured with two-photon imaging. Embedded sections were deparaffinized and hydrated prior to antigen retrieval and placement in blocking solution. Next, primary antibodies were sequentially applied upon removal of blocking solution at the following dilutions and incubation times: CD86 – 1:100 for 30 min, mCherry – 1:500 for 10 min, and CD206 – 1:1,000 for 5 min. Secondary antibodies were then added following each primary antibody incubation using rat and rabbit secondary antibodies. The following staining dyes were added after secondary antibody washes at 1:100 dilution for 10 min: CD86 – Opal-dye 520, mCherry – Opal-dye 570, CD206 – Opal-dye 620. Finally, stained sections were incubated in DAPI for 5 min at room temperature for nuclear labeling and mounted on coverslips for imaging. Imaging was performed at 20× using a Vectra multispectral imaging system (PerkinElmer) and a spectral library was generated to separate spectral curves for each of the fluorophores. Resulting images were analyzed using Nuance and inForm software (PerkinElmer).

### Statistical Analysis

Mann–Whitney statistical tests for non-parametric, unpaired comparisons were performed to assess differences in metabolic autofluorescence variables in dermal and tumor tissues. Results are represented as violin plots showing mean ± 95% CI or stacked bar plots showing mean ± SEM plotted in GraphPad. Heatmaps representing percent coefficient of variation calculations (measurement standard deviation divided by measurement mean multiplied by 100) for each autofluorescence variable per tissue type were also generated in GraphPad.

## Results

Representative images of optical redox ratio, NAD(P)H τ_m_, and FAD τ_m_ depict qualitative differences in the metabolic activity and spatial organization of dermal macrophages and tumor-infiltrating macrophages (Figure 2B). Macrophages (mCherry+ cells segmented with geodesic reconstruction) localize around hair follicles in the ear dermis, consistent with previous observations (Palero et al., 2007). Conversely, tumor-associated macrophages are randomly distributed across the tissue (Figure 2B). These results indicate that metabolic autofluorescence imaging can visualize metabolic differences in macrophages *in vivo* with single-cell resolution across multiple tissue types.

**FIGURE 3:**
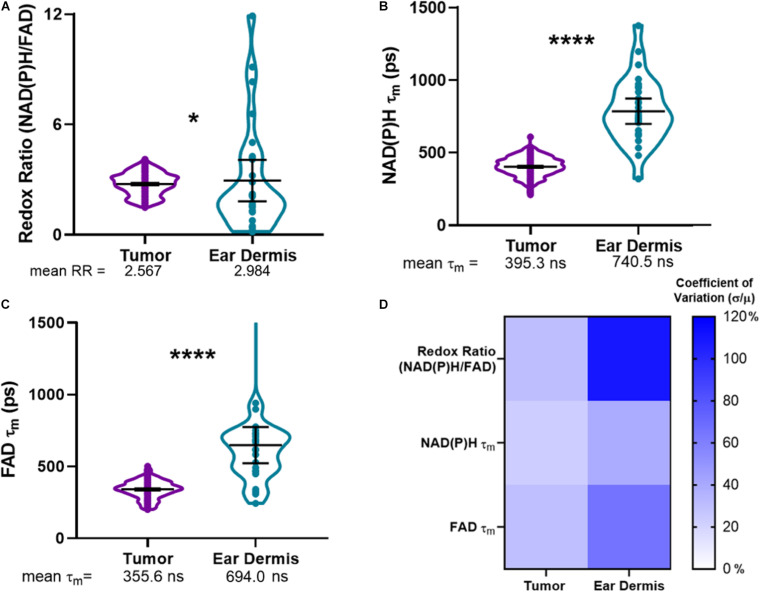
Metabolic autofluorescence resolves differences between tumor and dermal macrophages. **(A–C)** Violin plots show the full distributions of optical redox ratio (RR), NAD(P)H τ_m_, and FAD τ_m_ measured from individual macrophages in tumor and dermal tissues. Mean ± 95% confidence intervals for each measurement are represented by thick black lines and error bars, respectively, overlaid on the violin plots. **(A)** Quantitative single-cell analysis of redox ratio reveals that tumor-infiltrating macrophages are more oxidized than non-malignant dermal macrophages (2.567 ± 0.53 tumor RR vs. 2.984 ± 0.905 dermis RR). Additionally, mean lifetimes (τ_m_) of **(B)** NAD(P)H and **(C)** FAD are lower in tumor-infiltrating macrophage populations compared to non-malignant dermal macrophages. (*, ****p < 0.05, 0.0001; *n* = 4 mice, 836 cells – tumor; 52 cells – ear dermis). **(D)** Heatmap of coefficients of variation for autofluorescence variables demonstrate macrophage heterogeneity across both tissues, with the greatest variation observed across ear dermis macrophages. Percent coefficients of variation were calculated as the measurement standard deviation (σ) divided by the measurement mean (μ) multiplied by 100 for each variable and tissue type.

Using the composite imaging and single cell segmentation shown in Figure 2, quantitative analyses of the full data set for all mice enables comparisons of tumor and dermal macrophage autofluorescence *in vivo* (Figure 3). Tumor-infiltrating macrophages exhibited a small but significant decrease in optical redox ratio compared to macrophages within dermal tissue (Figure 3A), consistent with a metabolic preference for oxidative phosphorylation (Qian and Pollard, 2010; Panni et al., 2013). Furthermore, dermal macrophages displayed higher NAD(P)H τ_m_ and FAD τ_m_ compared to tumor-infiltrating macrophages (Figures 3B,C). The distributions of redox and lifetime values represented by the violin plots reveal notable metabolic heterogeneity in macrophages within both tissue types (Figures 3A–C). Dermal macrophages demonstrate greater metabolic heterogeneity compared to tumor-associated macrophages, as shown in the violin plots and coefficients of variation for all autofluorescence variables (Figure 3D). Macrophage variability is highest for the optical redox ratio, regardless of tissue type (CV: 30.37% – tumor; 108.05% – ear dermis). Ultimately, these quantitative trends illustrate the sensitivity of metabolic autofluorescence imaging to heterogeneous macrophage populations *in vivo*.

**FIGURE 4:**
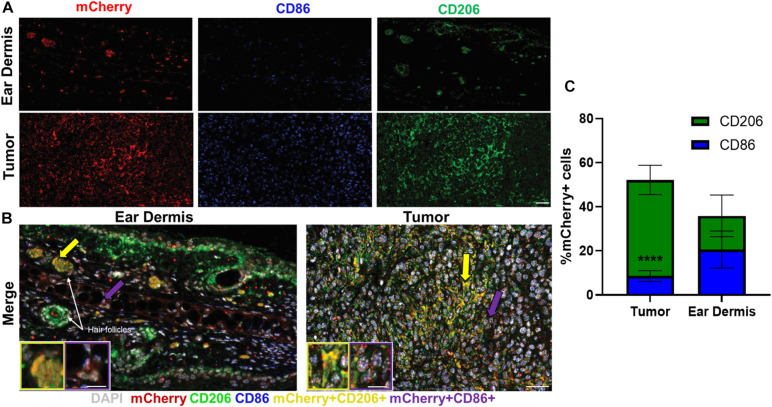
Immunofluorescence staining reflects heterogeneity in macrophage phenotypes across tissue types. Representative immunofluorescence images of resident macrophages in mouse ear dermis and infiltrating macrophages within mouse Panc02 pancreatic tumors. **(A)** Images of individual mCherry (red), CD86 (blue), and CD206 (green) channels visualize distribution of macrophage-specific markers in ear dermis (top) and tumor (bottom). **(B)** Merged images identify multiple macrophage phenotypes within both tissues. Inlays represent magnifications of cell areas indicated by yellow (left inlay) and purple (right inlay) arrows in original merged images. DAPI – nuclei, gray; CD86/mCherry co-localization – M1-like macrophage, purple; CD206/mCherry co-localization – M2-like macrophage, yellow. Purple arrows point to M1-like staining; yellow arrows point to M2-like staining. Scale bar, inlays = 15 microns, composite image = 50 microns. **(C)** Quantification of CD206+ mCherry+ macrophage and CD86+ mCherry+ macrophage proportions for both tissue types reveals a greater ratio of CD206+ to CD86+ macrophages infiltrating in tumor tissue than dermal tissue (****p < 0.0001 CD206+ vs. CD86+), demonstrating greater macrophage heterogeneity in dermal tissue (mean ± SEM; *n* = 4 mice, 22 ear dermis FOVs, 24 tumor FOVs).

Immunofluorescence was used to confirm the heterogeneity observed with *in vivo* autofluorescence imaging (Figure 4). Collectively, dermal tissue yielded much fewer total macrophages (mCherry+ cells) than tumor tissue, supporting the fewer number of macrophages imaged in the dermis compared to the tumor with *in vivo* autofluorescence imaging (Figure 4A). Macrophages positive for CD206, commonly associated with M2-like macrophages that prefer oxidative metabolism were abundant in tumors (Figure 4A). Significant CD206 staining was also observed for dermal macrophages but yielded much sparser expression than tumor-associated macrophages (Figure 4A). These CD206+mCherry+ macrophages (yellow, Figure 4B inlay) were also more prevalent surrounding hair follicles (white arrows), similar to the localization observed in metabolic autofluorescence images. Despite notable CD86 expression in tumor-associated macrophages, limited numbers of CD86+mCherry+ macrophages (purple, Figure 4B inlay) were detected across either tissue type. These qualitative observations are apparent in the quantification of relative proportions of CD86+ mCherry+ or CD206+ mCherry+ cells from immunofluorescence images (Figure 4C and Supplementary Figure 2). Tumor-infiltrating macrophages were observed to be primarily CD206+ (52.2% of mCherry+ cells vs. 8.5% CD86+ mCherry+ cells, *p* < 0.0001; Figure 4C), while dermal macrophages exhibited a more heterogenous mixture of CD206+ and CD86+ phenotypes (35.9% of CD206+ mCherry+ cells vs. 20.6% CD86+ mCherry+ cells, *p* > 0.05; Figure 4C). Note that some mCherry+ cells do not stain for CD86 or CD206 [e.g., non-M1/M2-type macrophages (Aras and Zaidi, 2017)], so the total percentage in Figure 4C does not reach 100%. Overall, immunofluorescence staining of excised mouse ear dermis and tumors confirmed that heterogeneous macrophage phenotypes contribute to the heterogenous macrophage autofluorescence in both dermal and tumor tissues. These immunofluorescence data also support greater heterogeneity in dermal macrophage phenotype compared to tumor macrophage phenotype, consistent with autofluorescence imaging.

## Discussion

This study establishes metabolic autofluorescence imaging as an effective approach for non-destructive, *in vivo* assessment of macrophage metabolism across normal and cancerous tissue in immunocompetent mouse models. Distinct tissue microenvironments have been previously shown to alter macrophage phenotype and function, providing innate perturbation of macrophage metabolism *in vivo* (Stout et al., 2005). Mouse ear dermis served as an optimal site to measure non-diseased tissue for comparison with engrafted tumors in a single animal, without the need for multiple surgeries. Autofluorescence imaging enabled quantitative assessment of metabolic heterogeneity within infiltrating macrophage populations across these tissues. Standard immunofluorescence confirmed the observations from metabolic autofluorescence imaging of macrophages within both dermal and tumor tissues. Collectively, these findings support the use of *in vivo* metabolic autofluorescence imaging to study relationships between macrophage behavior and the local tissue microenvironment.

The combination of intravital autofluorescence imaging and reporter mouse models ensures that the cells of interest (i.e., macrophages) can be accurately identified *in vivo.* Here, we used a previously established macrophage reporter model generated from crossbreeding Lyz2-Cre mice and mice harboring a floxed mCherry under the Csfr1 promoter (Schreiber et al., 2013; Hoffmann and Ponik, 2020). These mice express mCherry restricted to blood-circulating monocytes and those that have infiltrated into tissue becoming macrophages (Das et al., 2015). Previous studies injected fluorescently labeled antibodies into tissues to identify macrophages and then co-registered autofluorescence signals within these antibody-labeled macrophages *in vivo* (Szulczewski et al., 2016). This approach was limited by issues with *in vivo* antibody delivery, non-specific antibody binding, and spectral overlap between the fluorescent label and FAD autofluorescence. The use of fluorescent reporter mice in the current study provided macrophage-specific labels while circumventing these issues of antibody delivery and non-specific binding. The mCherry reporter used in the current study also did not interfere with NAD(P)H and FAD autofluorescence, similarly observed in previous *in vivo* autofluorescence studies (Hoffmann and Ponik, 2020; Miskolci et al., 2020). Identifying macrophage-specific autofluorescence features without fluorescent reporters remains challenging to due to substantial macrophage heterogeneity *in vivo*. However, this model could serve as a basis for cultivating reference *in vivo* datasets to train machine learning classifiers for unlabeled macrophages (Rostam et al., 2017; Christiansen et al., 2018; Pavillon et al., 2018). Overall, the combination of macrophage-specific mCherry reporter mice and two-photon autofluorescence imaging provide a convenient method to monitor macrophage behavior *in vivo*.

Macrophages traffic to specific tissues and undergo phenotypic switching in response to tissue-secreted biochemical signals (Aloysius et al., 2020). Dermal macrophages comprise a large proportion of cells (∼60%) in the mouse ear dermis, primarily of glycolytic, M1-like phenotype (Dupasquier et al., 2004; Italiani and Boraschi, 2014). The increased optical redox ratio of dermal macrophages reflects this abundant functional phenotype in the mouse dermis (Figure 3A). The increased optical redox ratio and FAD τ_m_ observed for dermal macrophages (Figures 3A,C) are also consistent with increased optical redox ratio and FAD τ_m_ in previous studies of 2D mouse macrophage cell lines stimulated to glycolytic, M1-like phenotypes (compared to naïve and M2-like macrophages) (Heaster et al., 2020). Similarly, macrophages are highly abundant in mouse pancreatic tumors (Sindrilaru et al., 2011; Ino et al., 2013). However, tumors commonly promote immunosuppressive function and oxidative metabolism in infiltrating macrophages to promote tumor survival, associated with M2 phenotype and function (Qian and Pollard, 2010). This reported preference for oxidative metabolism in tumor-infiltrating macrophages agrees with our measured decrease in optical redox ratio and FAD τ_m_ in tumor-infiltrating macrophages compared to dermal macrophages (Figures 3A,C). Our autofluorescence measurements in pancreatic tumor-infiltrating macrophages (Figures 2, 3) are also comparable to previous studies that show low NAD(P)H τ_m_ and high FAD intensity in tumor-infiltrating macrophages *in vivo* (Szulczewski et al., 2016). Small differences in absolute lifetime values reported here and in the previous *in vivo* autofluorescence study (Szulczewski et al., 2016) may be attributed to differences in tumor site (breast vs. pancreatic cancer) and tumor model (spontaneous vs. engrafted), which are known to alter cell autofluorescence (Heaster et al., 2020). Overall, these results demonstrate that metabolic autofluorescence imaging can detect heterogenous macrophage populations across normal and cancerous tissue based on unique macrophage metabolic activities.

Finally, phenotypic and functional heterogeneity captured from metabolic autofluorescence was confirmed with immunofluorescence staining of known macrophage markers. Diversity in metabolic autofluorescence was observed for both tumor-infiltrating macrophages and dermal macrophages (Figure 3D). Immunofluorescence confirmed this diversity, as mixed CD206 and CD86 expression was observed for both tumor-infiltrating macrophages and dermal macrophages (Figure 4). This heterogeneity is consistent with previous studies of tumor-associated macrophage diversity measured with flow cytometry in this tumor model (Biswas and Mantovani, 2012; Urs, 2019). Similarly, previous studies have measured diverse macrophage populations from immunofluorescence characterization of mouse skin comparable to the CD86/CD206 staining observed for ear dermis tissue (Sindrilaru et al., 2011; Lee and Prisby, 2017). This agreement with immunofluorescence supports autofluorescence imaging of NAD(P)H and FAD as a label-free *in vivo* method to monitor spatial and temporal variations in both macrophage metabolism and phenotype.

Here, we have shown that intravital metabolic autofluorescence imaging can resolve dynamic macrophage function within native tissue microenvironments, confirmed with fluorescent reporter mouse models. This provides an attractive method to assess macrophage behavior throughout disease progression and in response to treatment, which could aid in new drug development to target macrophages in cancer, wound healing, and a variety of other diseases. While autofluorescence FLIM alone is not sufficient to specify a biological mechanism, the *in vivo* macrophage imaging techniques described in this paper can be used to guide independent measurements of metabolism. The methods described here are also amenable to time-course measurements of macrophage movement and autofluorescence over hours and days within the same animal *in vivo*. Longer-term studies require alternative procedures (e.g., specialized vital regulation and imaging windows) though could provide further insight into the evolution of macrophage function *in vivo* (Ingman et al., 2006; Szulczewski et al., 2016; Entenberg et al., 2017; Dawson et al., 2021). These methods could also identify novel approaches to alter tissue state to influence macrophage function. Ultimately, this imaging approach could elucidate the dynamic role of tissue-specific macrophage populations *in vivo*.

## Data Availability Statement

The raw data supporting the conclusions of this article will be made available by the authors, without undue reservation.

## Ethics Statement

The animal study was reviewed and approved by Institutional Animal Care and Use Committee, the University of Wisconsin–Madison.

## Author Contributions

TH designed the experiments, collected the data, designed and performed the analysis, interpreted the data, generated the figures, and drafted the manuscript for this study. AH assisted in performing the analysis, interpreting the data, and editing the manuscript. PS and MC helped to design the experiments and analysis, interpreted the data, and edited the manuscript. All authors contributed to the article and approved the submitted version.

## Conflict of Interest

The authors declare that the research was conducted in the absence of any commercial or financial relationships that could be construed as a potential conflict of interest.
