# Supplementary material for: Intravital Metabolic Autofluorescence Imaging Captures Macrophage Heterogeneity Across Normal and Cancerous Tissue
Source: Front Bioeng Biotechnol. 2021 Apr 20;9:644648. doi: 10.3389/fbioe.2021.644648 (PMC8093439; doi:10.3389/fbioe.2021.644648)
Supplement: Supplementary file 1 [file Data_Sheet_1.DOCX]

Supplementary Material

# Supplementary Figures


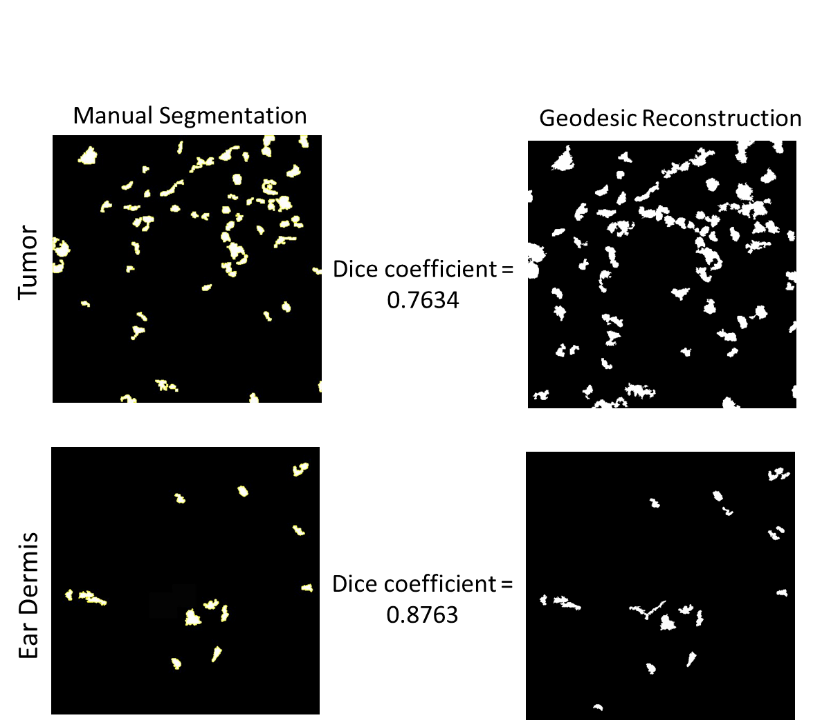


**Supplementary Fig. 1. Comparison of manual and automated segmentation of *in vivo* macrophage images.** Representative cell masks and outlines from manual segmentation (left) and automated segmentation via geodesic reconstruction (right) from matching fields-of-view in tumor and ear dermis. Dice coefficient provides a quantitative metric of similarity between manually- and automatically-segmented images where a value of 1 represents identical segmentation.


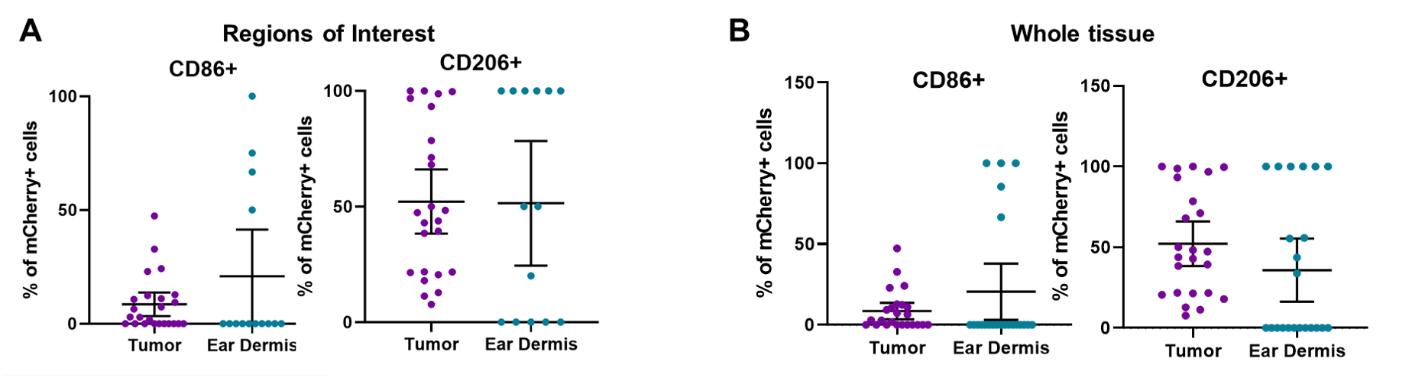


**Supplementary Fig. 2. Quantification of immunofluorescence markers show distribution of macrophage subpopulations present across tissue types.** Distribution of quantified colocalized CD86+/mCherry+ and CD206+/mCherry+ stained cells are shown for A) manually-selected regions of interest and B) whole tissue sections of mouse tumor and ear dermis. Regions of interest were selected to identify hair follicles in ear dermis. Quantified data distributions show similar infiltration of M2-like macrophages (CD206+) in tumor tissue and around dermal hair follicles, while follicles suggest mild differences in accumulation of M1-like (CD86+) macrophages (mean ± 95% CI; n= 24 tumor FOVs, 14 hair follicle FOVs). Conversely, whole tissue quantification proposes a modest increase in M2-like macrophages (CD206+) found infiltrating tumor tissue, while ear dermis tissue have comparable accumulation of M1-like (CD86+) and M2-like (CD206+) macrophages (mean ± 95% CI; 24 tumor FOVs; 115 cells, 22 ear dermis FOVs).
